# Current Analytical Methods and Research Trends Are Used to Identify Domestic Pig and Wild Boar DNA in Meat and Meat Products

**DOI:** 10.3390/genes13101825

**Published:** 2022-10-09

**Authors:** Małgorzata Natonek-Wiśniewska, Agata Piestrzynska-Kajtoch, Anna Koseniuk, Piotr Krzyścin

**Affiliations:** Department of Animal Molecular Biology, National Research Institute of Animal Production, 32-083 Balice, Poland

**Keywords:** pig, wild boar, species identification, genetic markers, genetic methods

## Abstract

The pig, one of the most important livestock species, is a meaningful source of global meat production. It is necessary, however, to prove whether a food product that a discerning customer selects in a store is actually made from pork or venison, or does not contain it at all. The problem of food authenticity is widespread worldwide, and cases of meat adulteration have accelerated the development of food and the identification methods of feed species. It is worth noting that several different molecular biology techniques can identify a porcine component. However, the precise differentiation between wild boar and a domestic pig in meat products is still challenging. This paper presents the current state of knowledge concerning the species identification of the domestic pig and wild boar DNA in meat and its products.

## 1. Introduction

**Figure 1:**
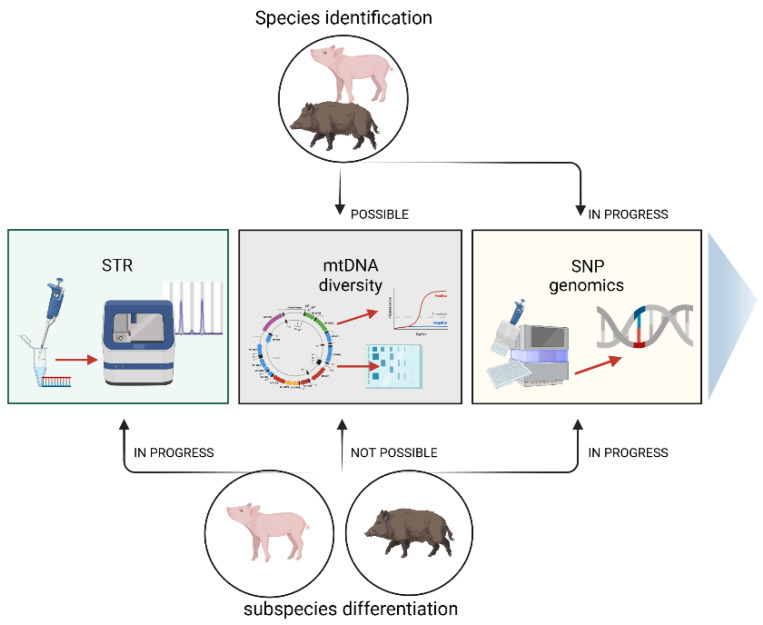
Methods of species identification of domestic pigs and wild boars and distinguishing between both subspecies.

The pig is believed to be one of the most important livestock species for humans. It is an excellent animal model for studying the molecular background of several human diseases [1], and above all, it is a meaningful source of global meat production. Pork is widely used in the food industry, but one problem is determining the food’s authenticity, especially in terms of the species. Meat adulteration involves replacing or partially replacing high-value meat with cheaper quality meat [2], which involves economic, quality, safety, and socio-religious issues [3]. Together with pork, products may introduce veterinary drugs banned from the food chain, such as ractopamine, which is forbidden in many countries [4,5]. Moreover, undeclared pork may be contaminated with harmful microorganisms, such as the endoparasites Toxoplasma and Trichinella [6]. The presence of pork in food may also conflict with religious and cultural practices. Thus, the fraud of undeclared pork in food can affect consumer trust in the meat industry. Because of such illegal food practices, many governments have enacted laws prohibiting similar fraud. To ensure existing regulations are followed, laboratories worldwide have designed and developed various methods to identify animal species in food products, including methods to detect porcine components. According to scientific reports, efforts have also been made to provide a cost-efficient diagnostic tool for distinguishing wild boar (*Sus scrofa*), domestic pig (*Sus scrofa domestica*), and their hybrids (Figure 1). This is not easy, as the pig and the wild boar are evolutionarily closely related [7], and thus share many genetic markers. However, the identification methods have changed over time, accompanied by the development of innovative techniques that are now based on DNA testing. This work aims to present the current state of knowledge concerning the species identification of the domestic pig and wild boar DNA in meat and its products.

Possibilities of species identification of pigs and wild boars (top of the figure) and distinguishing between both subspecies (bottom of the figure) using different molecular techniques.

## 2. Pig and Wild Boar Phylogenetics

A considerable number of animal breeds on Earth originated from diverse wild ancestors which, after being domesticated, exhibit a large phenotypic variety [8]. In the study by Groenen et al. [7], a comprehensive analysis of the phylogenetic background of the subspecies structure of wild boars and pigs was provided. The genomes of wild boars and domestic pigs from Europe and Asia revealed separate European and Asian lineages. The study also gave an insight into the history of population size changes. Both lines of wild boars diverged about 1.6–0.8 Myr (million years) ago, resulting in alleles divergence in the millions of loci [7]. Wild boars are the ancestors of modern domestic pigs, and it is believed that multiple domestication events occurred separately for species in Asia and Europe [7,9].

After domestication, many incidents of the admixture of domestic populations with local wild boars occurred because both species are found in the same habitat [9]. Moreover, there are farms (e.g., in Bulgaria and Sardinia) where pigs are kept in semi-wild conditions. It is highly likely that domestic pigs sporadically crossed with wild boars [10]. The frequent hybridisation of these two breeds was found in the studies on the European population [10,11,12,13]. Studies of the *MC1R* (melanocortin 1 receptor) gene in the Polish population of wild boars also proved the existence of admixed genotypes [14,15]. Crossbreeding domestic pigs with wild boars is documented and has served hunting purposes, better meat production, better taste, and reduced aggressive behaviour [11,12]. However, crossbreds have a high risk of pathogen transmission (e.g., ASF, African Swine Fever), which is the primary reason crossbreeding farms are under strict legislation in the European Union [12]. It is highly likely that some wild boars farmed in Poland were released or escaped from the farms and became a potential source of genome admixture in the wild population. Other studies have also suggested such a scenario [11,12,14]. The domestication of the pig and the mixing of domestic pig and wild boar populations cannot be ignored, as they provide the background to the problem of distinguishing between these subspecies.

## 3. Authentication of Meat from Domestic Pig

The authentication of domestic pig meat is mainly based on mitochondrial DNA (mtDNA). The well-known sequences of the mitochondrial genome allow comparison of the porcine mtDNA with the genome of any species. The advantages of mitochondrial DNA over genomic DNA are due to its properties. The mitochondrial genome is well understood and described in many databases (GenBank, EST, GSS). It is also species-specific and is present in many copies in every organism cell. Moreover, mtDNA is resistant to unfavourable factors, such as high temperature or pressure. This last feature is essential because it allows the identification of DNA in preserved meat products, the production of which involves thermal processing. Properties of mtDNA translate into biological specificity and high sensitivity of pork identification methods, which is very important when investigating highly processed products such as sausages, canned pork, pork fat, or gelatine. However, to date, it is impossible to distinguish pork from venison based on mtDNA variability only.

The heat treatment of food causes fragmentation of the DNA. This may reduce the chance of extracting good quality DNA sufficient for further analysis. In the literature, we can find many reports on the influence of individual types of heat treatment on DNA quality. Traditional cooking, heating in a microwave [16], or processing pork for canned meal influence the amount of DNA obtained, and the isolate rate remains at a reasonable level. In contrast, DNA extracts from gelatine or meat meal had much worse properties. In the first case, the quantity and quality of DNA described by the absorbance ratio had the values of 400–600 ng/µL and 1.7–1.9, respectively. However, only 15–145 ng/µL were obtained for gelatine or meat meal, with a purity often below 1.6 or above 1.9. These are the difficulties any laboratory faces in food product species identification.

The obtained DNA can be analyzed by many methods. Most of them are based on the PCR reaction, which can be used in several variants such as classical PCR, real-time PCR, sequencing, DNA barcoding and NGS (next-generation sequencing). The advantages and limitations of these methods are presented in Table 1.

## 4. Methods Used for Species Identification—Possibilities and Application

### 4.1. PCR

Methods based on classical PCR are used mainly for qualitative analysis of a specific species or group of different species. They are very useful when we want to check the potential presence of swine DNA. They provide the highest analysis sensitivity of all DNA-based methods. Qualitative identification of porcine DNA is a perennial research problem, yet it still enjoys unabated interest. However, research tendencies have changed over the years. Twenty years ago, all methods were based on the analysis of long DNA fragments. Now, short counterparts, often below 100 bp, are preferred. These changes are related to the more excellent suitability of short amplification products for analysing processed samples.

### 4.2. PCR RAPD

The random amplified polymorphic DNA (RAPD) technique is based on DNA amplification, with a short arbitrary primer that amplifies multiple fragments of the analyzed genome. It is followed by separating obtained DNA fragments (based on their sizes) using gel electrophoresis. PCR-RAPD for species identification is most useful for rapid qualitative analysis. In the literature, it was described as a helpful tool for differentiating domestic animals, including pigs [38]. The identification of particular species DNA in meat samples using a random amplified polymorphic DNA (RAPD) technique because of numerous obstacles is rarely used for commercial samples [38,39].

### 4.3. PCR-RFLP

The basis of the PCR-RFLP (Restriction Fragment Length Polymorphism) reaction is the amplification of a large amplicon homologous for several species using a standard pair of sequence-specific primers. The large amplicons are then digested using one or more restriction enzymes into shorter reaction products. The method is efficiently implemented to screen for several species’ DNA in one sample [40,41], even for gelatine [42]. Because of its properties, the technique has been successfully implemented for the complex analyses of animal groups (mammals, birds, or being a potential ingredient of a product, even gelatine) and then their specific representatives. Restriction enzyme analysis to detect pork has been performed frequently over the past several years. It enables us to simultaneously distinguish different species, including farm animals [40,43], and sometimes in combination with wild or companion animals [44,45]. Thus, despite many limitations (Table 1) and newer techniques, the restriction enzyme method is still used to determine pork in food products.

### 4.4. Real-Time PCR (Quantification and Qualification)

Real-time PCR is an easy method that allows qualitative and quantitative measurement of the species composition in any product. This technique is probably the most widely used for pork identification of all PCR-based techniques. Depending on the probes or dyes used, results occur in a shorter time than the classic PCR. In quantitative analysis, more accurate (accuracy, precision) results are obtained using probes (Taqman, TAMRA, Dabcyl, BHQ). The analysis is useful in controlling the declaration of the species composition in gelatine [46] and processed foods [29]. In both cases, the standard deviation of C_T_ and DNA concentration below 4% were demonstrated. The dyes (SYBR Green, EvaGreen) can also be used for a quantitative reaction, but the obtained results have a more significant measurement error [47]. On the other hand, in the qualitative determinations, the method with dyes works perfectly [48,49]. Currently, much emphasis is placed on the possibility of declaring a product vegan or kosher. A natural consequence of such a declaration should be the ability to identify falsifications at an extremely low level. A recent study indicated that the sensitivity of the real-time PCR method is improving. It is already possible to identify adulteration at 10 pg [47] or a concentration of 0.001% [48].

### 4.5. Digital PCR

Digital PCR (dPCR) is an alternative, sensitive, and specific method but does not rely on a standard curve. The basis of dPCR is to quantify the absolute number of targets present in a sample using limiting dilutions, PCR, and Poisson statistics. Because the habitat of reaction is divided into many smaller ones and endpoint amplification is less susceptible to competition between targets, this technique can detect small numbers of targets. It is useful whenever we need to perform quantitative determinations. Currently, this technique is increasingly more accurate than qPCR, owing to the lack of dependence on matching the type of reference material to the tested samples. It is described as suitable for usage by competent food safety authorities to verify compliance with the labelling of meat products, and to ensure quality and safety throughout the meat supply chain [50]. The studies found that the sensitivity of the method was very high—the lowest concentration of pork DNA, for which at least 95% of the replicates were positive, was 0.1 pg/μL [50]. As mentioned, the method shows the absolute result expressed in DNA copies. However, for practical purposes, there are ways of converting the result into the classical concentration of a component of a given species. Based on tests of 20 sausage samples carried out in six laboratories, it has been shown that the method can be used to evaluate meat in mixed meat products, with the highest accuracy and precision compared to the results generated by real-time PCR [31].

### 4.6. LAMP

Loop-mediated isothermal amplification (LAMP) is a rapid, economical and species-specific DNA-based assay for the authentication of animal species. The technique amplifies the animal mitochondrial D-loop region by isothermal amplification. In recent years, many laboratories have been working on developing this technique because of the shorter and less expensive hardware requirements compared to the PCR test, and the parameters sufficient for its use in the testing of commercial samples. The assay can detect pork to the level of 100 fg admixture, and the DNA detection limit was 0.0001% [51].

Cross-amplification of related species like cattle, buffalo, sheep, goat, and chicken was excluded by incorporating their DNA in the reaction assay [51,52]. It can also be integrated into compact lab-on-a-chip devices to develop micro-total analysis systems [51,53]. An essential feature of the research is the ability to analyze processed meat [52]. This makes LAMP a serious competitor to real-time PCR methods in pork identification in typical food samples.

### 4.7. DNA Barcoding and NGS

DNA barcoding generally relies on the use of a specific genetic target. The conventional DNA barcoding target is usually a ~650 bp region of the mitochondrial COI gene. This method is one of the most basic and oldest methods of species identification. It is mainly used for the analysis of cooked food samples. A study revealed that the rate of mislabelling for pork sausage was 21% [36,54].

The recent and fast development of benchtop next-generation sequencers has made NGS technologies highly applicable for meat speciation, including pork [37]. Most genome-wide association methods have been introduced using Porcine SNP Beadchips (Illumina, Inc., San Diego, CA, USA) and whole genome resequencing. These methods allow for the analysis of several SNP markers, CNVs, indels and STRs in a single reaction run. Studies have indicated that the sensitivity of this method is 1% of the total DNA [55].

Connecting both techniques (barcoding and NGS) improves the performance of analysis in comparison to traditional barcoding. Therefore, they can be serious candidates for species identification in the near future. Recently, the utility was presented for extracts from muscle meat, and DNA admixtures from model sausages [56] and commercial lamb sausages [57]. The observed limit of detection (LOD) was on the level of 0.1% [56] and allowed for the detection of a few adulterations in porcine DNA from processed food [57,58]. The NGS-based methods can also distinguish domestic pigs and wild boars.

## 5. Genetic Markers Used for Pig and Wild-Boar Distinguishing

Molecular techniques to identify animal species components in human food and animal feed use different genetic markers based on genomic or mitochondrial DNA. There are cost-effective and easily performed tests for DNA identification of cattle, horse, and poultry in food and animal feed [17,59]. Some of the methods used for pigs were discussed above. A reliable method for the differentiation of both subspecies would impact biodiversity, food fraud cases, detection of illegal hunting procedures, and zoonosis prevention. However, whether genomic DNA markers can be useful for analysis performed on DNA extracted from highly processed food products remains a subject of study.

### 5.1. Mitochondrial DNA Genome Loci

Mitochondrial sequences have been widely studied in the context of phylogenetics in many species [60,61,62], but using mtDNA only for distinguishing wild boar from domestic pig is unreliable. A different approach is needed. Fajardo et al. [63] conducted studies on wild boars and commercial pig breeds differentiation based on combining both nucleus (melanocortin 1 receptor, *MC1R*) and mitochondrial DNA (*D-loop*) analysis. The analysis of the *MC1R* gene proved to be more effective for species identification based on the polymorphism of mitochondrial DNA [63].

### 5.2. Melanocortin 1 Receptor Gene (MC1R)

The animal’s coat colour is a visual feature that reflects changes in environment and breeding selection. It is a trait that phenotypically differentiates pigs and wild boars. Wild animal forms are characterised by a dark fur colour, while domestic species have pale coat colours [61]. Several genes have been identified as associated with skin and hair pigmentation. *MC1R*, along with the *ASIP* gene, regulates the synthesis of two dyes: eumelanin (brown to black) and pheomelanin (crema to red) in melanocytes [64]. Both genes express an epistatic interaction [65,66]. An *MC1R* allele (E + allele, also called “wild” allele) unique to the wild boar’s population was identified by Kijas et al. [67]. Most likely, owing to selection during the breeding work, individuals with the “wild” allele were lost. Implementing PCR-RFLP and real-time PCR protocols to identify four polymorphic *MC1R* loci gave inconsistent results. In Greece, a population of pigs and wild boars was distinguished using this marker [68]. However, further studies revealed that the “wild” allele was also present in some domestic breeds, and “domestic” alleles were found in wild boar samples [11,14,69].

### 5.3. Nuclear Receptor Subfamily 6, Group A, Member 1 (NR6A1)

Owing to increasing meat efficiency, domestic pig breeds are characterised by more vertebrae. This is another trait that differentiates wild boars and European commercial pigs. In the wild boar, the number of vertebrae is 19 and in pig breeds it is 21–23. A proline to leucine substitution at codon 192 (*p.Pro192Leu*) in the nuclear receptor subfamily 6, group A, member 1 (*NR6A1*) gene was shown to be the most likely causative mutation underlying the QTL (Quantitative Trait Loci) [70].

Since neither *MC1R* nor NR6A1 studies as single markers proved to be an effective tool to differentiate both species, the combination of both genes was studied. In general, the combination of single nucleotide polymorphisms in *MC1R* and *NR6A1* genes was tested using real-time PCR [11,71] or multiplexed using the SNaPshot Multiplex System method [72]. The results were promising.

### 5.4. Short Tandem Repeats (STRs)

Short tandem repeats (STRs, also known as microsatellites) are genetic markers that are commonly used in parentage testing and verification [73]. These markers are specific for the species, so they seemed good candidates to distinguish wild boar and pig. The hybridisation between pigs and wild boars based on STRs and *MC1R* was studied by Nikolov et al. [74]. STRs combined with SNPs in *MC1R*, *NR6A1* genes or mitochondrial D-loop have also been analyzed [10,75].

Bayesian clustering based on 10 STRs and mitochondrial D-loop polymorphic loci indicates that the wild and domestic forms are not divergent. Wild boars and domestic pigs share the most common mtDNA haplotypes, and microsatellite alleles weakly resolve both groups when examining a phylogenetic tree [10]. Nikolov et al. [74] proved the introgression of wild boar to Balkanian domestic pig breeds by studying *MC1R* genotypes and 10 STRs. In the population from Balkanste, the introgression in wild boar is more apparent than the introgression in domestic pigs from Western Europe [74]. Lorenzini et al. [75] implemented the STRs protocol combined with *MC1R* and *NR6A1* to identify both subspecies for forensic purposes. In this case, STRs were introduced to group individuals into the parental population. However, this marker type does not efficiently identify recent hybrids. STRs alone identify lower hybrids than genotyping polymorphic loci, along with *MC1R* and *NR6A1* [75].

### 5.5. Other Potential Genetic Markers

As pigs and wild boars are closely related, and genetic introgression from domestic pigs into European wild boar has been proved [74,76], phylogenetically close taxa distinguishing requires introduction of high-throughput, SNP-based (SNP, Single Nucleotide Polymorphism) molecular techniques. A 70K SNP chip was used in a French study [13] to determine the timing of the hybridisation (s) and to check the domestic pig admixture in the wild boar population. The study revealed that among wild boars, 83% to 100% of animals had a genome of “wild” origin and local ancestry analyses showed adaptive introgression from a domestic pig. These results indicated that population admixture must be considered in studies on pig and wild boar distinguishing.

Some loci responsible for phenotypic features important in domestication were revealed by Rubin et al. [77] with the whole-genome resequencing method. They found 33 candidate selective sweeps representing 18 different loci. Among others were regions on chromosome 1 (*SSC1*) including the *NR6A1* gene (described above), on *SSC4* including the *PLAG1* gene, on *SSC8* including the *LCORL* gene, and *SSC13* overlapping the *OSTN* gene. The *PLAG1* gene was previously associated with human and cattle height [78,79], and the *LCORL* gene was connected to the body size of various species [80,81,82]. *OSTN* produce the osteocrin, which is an inhibitor of osteoblast differentiation [83]. Its expression levels differed between muscle fibre types [84] and were correlated with food intake [85]. In the same project, authors found 72 nonsynonymous substitutions in the coding sequences, with the difference in allele frequency between pig and wild boar (i.e., *NR6A1*, *CYCS*, *ACER1*, *HK2*, *KITLG*, *TCTE1*, *SERPINA6*, *SEMA3D*, and many others) three of which (in *NR6A1*, *CCT8L2* and *MLL3*) were colocalized with putative sweep regions [77]. All of these associations between putative sweeps and phenotypic differences could potentially be used to distinguish wild boars and domestic pigs.

Wilkinson et al. [86] used a Porcine 60K SNP chip to find signatures of diversifying selection between different pig breeds (traditional and commercial European pig breeds), and also compared the pig breeds with wild boar. Among others, the results showed high levels of differentiation between pig and wild boar genomic regions connected to bone formation (on *SSC1*), growth (on *SSC7*), and fat deposition (on *SSC7* and *SSCX*). The differentiated genomic region on chromosome 7 was the most extensive and contained such genes as *PPARD* and *CDKN1A* associated with fat deposition [87,88]. It was close to the *MHC* loci, which is known to be a crucial, vertebrate immunity complex. Wild boar-specific selection signatures in *SSC7*, with one of the regions in *SSC7* located close to the *PPARD* gene, were also observed by Muñoz et al. [89]. Fatness was one of the features under the strong selection in domestic pigs [90].

Seeking global patterns in pig domestication, Yang et al. [8] calculated the genome-wide fixation index (Fst) between domestic pigs and wild boars (from Asia and Europe, separately) based on a genome-wide single nucleotide polymorphism. Among genes located close to the top outlier, SNPs with the highest Fst values were ones associated with phenotypic features and traits that changed during domestication, such as growth (*SOX2-OT*), muscle development (*MSTN*), energy balance (*NMU*, *LEP*, *GSK3A*), social behaviour (*TBX19*, *PAFAH1B3*), reproduction (*ESR1*, *GNRHR*, *PATZ1*, *PLSCR4*), metabolism (*TMEM67*, *FOXA1*, *INSIG2*), central nervous system (*LRRC4*, *VEPH1*, *CDH9*), immune system (*LAIR1*), and even perception of smell (*Olfr466*) [8]. The polymorphisms linked to genes, candidates for domestication loci, could potentially be used as markers for pig and wild boar distinguishing.

Recently, Moretti et al. [91] proposed a method to select a reduced SNP panel for the traceability of pig breeds and wild boars. The panel allowed discriminating among all species and wild boars in their study, and even to distinguish hybrid crossing.

The methods and markers implemented to date for distinguishing both subspecies in meat and muscle are summarised in Table 2. Although some of the genetic markers seem promising, none of the above markers was tested on DNA isolated from meat products. Since the analysis of such samples requires a unique approach, as the DNA isolated from food products is fragmented, methods for genotyping mutations may generate false negative results. Further studies are needed to resolve that problem.

## 6. Conclusions

Pork identification in food is essential from an economic, quality, safety, and socio-religious points of view. Several methods can be used for this species identification, such as PCR, real-time PCR, PCR-RAPD, PCR-RFLP, Digital PCR, LAMP, DNA barcoding and NGS. Some methods (classical PCR and real-time PCR) have been used since the 1990s and are still being improved for the analysis of more processed food. There are also possibilities for distinguishing between pork and wild boar meat. However, further study is needed to establish reliably distinguishing methods.

According to us, the best hope for further development in this field of research is provided by simple and/or modern techniques. A simple method like PCR (classic or real-time) is sufficient for fast and cheap pork identification, while modern techniques are preferable owing to the undeniable advantages of accuracy (Digital PCR), low price (LAMP), high specificity (NGS), and high throughput (DNA barcoding and NGS). In our opinion, each of these techniques is meaningful and useful for strictly defined types of samples (raw/processed/canned food; one-species meat/mixed meat of different species) and problems we must resolve, such as species identification of pig or differentiation their subspecies.

## Figures and Tables

**Table 1:** Advantages and limitations of different methods used for Sus scrofa meat identification.

Technique/Method	Advantages	Limitations and Difficulties	References
PCR	Simple to develop and easy to conduct Can detect small amounts of DNA High specificity species identification Analysis of monoplexes or multiplexes depending on needs Low detection limit Ability to detect processed and heat-treated samples	No quantitation Lower specificity when amplicon length is short More difficult PCR optimisation in case of multiplex PCR Unequal amplification efficiency which causes variable sensitivity Contaminants could produce false-negative results Lower DNA yield for heat-treated samples	[17,18,19,20,21]
PCR RAPD	Detects multi-species, and no previous knowledge of DNA is required Can detect a high level of polymorphism (for example, between similar species) Costs per assay are low	Unable to differentiate species in mixtures Mistakes during the analysis of degraded or autoclaved materials Difficulties in obtaining reproducible results	[22,23,24]
PCR-RFLP	Species-specific restriction pattern	The results can be challenging to interpret in the case of entirely unknown species components of the studied object Inadequate for highly processed or meat mixtures	[25,26]
Real-Time PCR (quantification and qualification)	Possible quantitative result Very high sensitivity High sample throughput	Probe-based methods are expensive and time-intensive Dye-based methods are less accurate Quantification PCR requires appropriate reference material Precision of measure is different for different product types qPCR instruments are very costly HRM analysis requires HRM-capable real-time PCR machines and specialised software algorithms	[27,28,29]
Digital PCR	Better detection limit and accuracy	Little sample volume per reaction Small dynamic range if the number of partitions is limited The risk of falsely low quantification due to molecular dropout Restricted multiplex detection	[30,31]
LAMP	Rapid High specificity High amplification efficiency No need for thermal cycling Low operational cost	Less versatile Little to no multiplexing Less sensitive to inhibitors than PCR in case of complex samples. The method is more complicated than classical PCR owing to the presence of more primers	[32,33,34,35]
DNA barcoding and NGS	High sensitivity and specificity Single-step DNA sequencing and quantification by NGS High throughput detection	High-quality DNA necessary Long analysis time from DNA extraction to the finish of bioinformatics analysis Complex library preparation protocol for NGS Trace quantity of foreign DNA may cause inaccurate estimation of species Costly equipment to analyse and very highly skilled personnel	[36,37]

**Table 2:** The molecular techniques and markers implemented for identifying domestic pig and wild boars and their hybrids in meat or muscles.

Technique	Marker/Gene	Tested on Sample of		References
		Muscle	Meat	
PCR RFLP and real-time PCR	*MC1R*, *NR6A1*, mtDNA regions	yes	yes	[11,14,63,68,70,71,72]
PCR multiplex	STRs	yes	no	[10,75]

## Data Availability

Not applicable.
